# The Utility of Repeat Endoscopic Ultrasound-Guided Fine Needle Aspiration for Suspected Pancreatic Cancer

**DOI:** 10.1155/2010/268290

**Published:** 2010-12-28

**Authors:** Mark Nicaud, Wei Hou, Dennis Collins, Mihir S. Wagh, Shailendra Chauhan, Peter V. Draganov

**Affiliations:** ^1^Division of Gastroenterology, Hepatology, and Nutrition, Department of Medicine, College of Medicine, University of Florida, 1600 SW Archer Road, Room HD 602, P.O. Box 100214, Gainesville, FL 32610-0214, USA; ^2^Department of Epidemiology and Health Policy Research, University of Florida, Gainesville, FL 32611, USA

## Abstract

*Background*. The utility of repeat EUS in patients with suspicion for pancreatic cancer after non-diagnostic EUS-FNA study is not well established. *Aim*. Determine the accuracy of repeat EUS-FNA in patients with suspected pancreatic cancer and prior non-diagnostic EUS-FNA. *Methods*. Retrospective cohort study. *Results*. From 2002 to 2008 in our institution 28 patients underwent repeat EUS-FNA for suspected pancreatic cancer. Initial EUS showed a pancreatic mass in 24 (85.71%), no mass in 3 (10.71%) and possible mass in 1 (3.58%). FNA was performed and was negative for malignancy in all patients. Repeat EUS showed pancreatic mass in 27 patients (96.42%) and no mass in 1 (3.58%). FNA was performed in all patients and cytology was positive for malignancy in 6 (21.43%). Out of the 28 patients, 17 (60.71%) were eventually confirmed to have cancer. Overall repeat EUS-FNA correctly determined the true final status in 17 out of 28 patients providing sensitivity for the diagnosis of cancer of 35% (95% CI 14%–62%), specificity 100% (95% CI 72%–100%), and overall accuracy of 61%, (95% CI 28%–72%). *Conclusion*. Repeat EUS-FNA provides reasonable accuracy and may be worthwhile in patients with suspected pancreatic cancer who had had prior negative EUS-FNA.

## 1. Introduction

Pancreatic cancer caused 35,240 deaths in 2009 making it the 4th leading cause of cancer-related death in the United States [1]. The disease typically presents at a late stage given the lack of early detection methods and late manifestation of symptoms. The prognosis of pancreatic cancer is grim with 1- and 5-year-survival rates of 24% and 5%, respectively [1]. Surgical resection is the only potentially curative option, but because of the late disease presentation only 15%–20% of patients are surgical candidates. Therefore, accurate tissue diagnosis and staging is imperative prior to considering any surgical or palliative chemoradiation therapy. 

Endoscopic ultrasound (EUS) with fine needle aspiration (FNA) has been shown to be both safe and accurate in providing definitive diagnosis in patients with pancreatic cancer [2]. Since EUS-FNA is considered the gold standard for pancreatic cancer staging and tissue acquisition, the practicing physician is faced with the dilemma of how to manage patients who have high probability of having cancer based on clinical presentation but have a non-diagnostic EUS-guided FNA. A large multicenter study showed that EUS-FNA of solid pancreatic lesions confirmed malignancy in 71% of the patients [3]. Therefore, a substantial number of patients with pancreatic lesions remain with no definitive diagnosis despite attempted EUS-FNA, and currently there is no accepted unified management strategy for such patients. One approach is to repeat the EUS exam and reattempt FNA, but there is limited data to support such strategy. The aim of this study was to determine the accuracy of repeat EUS-FNA for tissue diagnosis in patients with suspected pancreatic cancer with an initial non-diagnostic EUS-FNA.

## 2. Methods

This is a retrospective review of prospectively collected data in a searchable electronic database. All patients who underwent more than one EUS-FNA in our institution for evaluation of suspected pancreatic cancer with initial non-diagnostic EUS-FNA were included in this analysis. Patients were excluded if tissue diagnosis was secured at the first EUS. The definition of a non-diagnostic EUS-FNA included cases in which FNA was attempted in patients with suspected pancreatic cancer, but the cytology results were negative for malignancy for one of the following reasons: adequate sample material was obtained, but cytology was negative for malignancy, less than the predetermined minimum of four FNA passes were made for any reason, and successful four FNA passes were carried out, but inadequate sample material was obtained.

All patients provided informed consent. EUS was done using the curvilinear echoendoscope (Olympus UC-30P, or UCT 140; Olympus America Inc, Center Valley, PA) as previously described [4]. Briefly, all cases were done using endoscopist-guided sedation using combination of meperidine or fentanyl with midazolam with the goal to achieve moderate sedation. With the patient in left lateral decubitus position, the echoendoscope was inserted, and the pancreas was indentified. Features of chronic pancreatitis were systematically documented as previously defined [5]. Patients with four or more features were considered to have chronic pancreatitis. Color Doppler was used to exclude intervening vascular structures and to choose a vessel-free needle track. All FNAs were performed utilizing Echo-tip 22 French gauge EUS needle (Cook Endoscopy, Winston-Salem, NC). Suction via the needle was applied in all cases. The FNA samples were evaluated immediately for adequacy by a cytopathology technician. The goal was to obtain at least four passes from the lesion unless cytological evaluation performed on site confirmed the presence of malignant cells. We utilized the final cytology reports for our analysis. Cytology was categorized as (1) benign/reactive, (2) atypical, (3) suspicious, or (4) malignant. The final diagnosis of pancreatic cancer was defined based on one of the following criteria: (1) positive cytology at the second EUS, (2) positive cytology or histology obtained by alternative means (e.g., surgical biopsy), (3) clinical progression judged by evolving local invasion or metastatic disease on followup computed tomography (CT) or magnetic resonance imaging (MRI), or (4) death from the disease within 12 months of initial presentation. Complications were defined and graded based on accepted consensus criteria [6]. Complications were assessed during the procedure and in the postprocedure recovery area and then by telephone call the day after the procedure. All detected complications were entered in a dedicated searchable electronic database. 

### 2.1. Ethics

The study was approved by the institutional review board (IRB) at the University of Florida, and all patients signed informed consent.

### 2.2. Statistics

Continuous variables were reported as means and standard deviation, while categorical variables were reported as proportions. Statistical analysis was performed by using SAS version 9.1 (SAS institute, Cary, NC). The sensitivity, specificity, positive predictive value, and negative predictive value of EUS 1 and EUS 2 were calculated. 95% confidence intervals of those statistics were also calculated for comparison [7].

## 3. Results

From January 2002 to November 2008, total of 3895 patients underwent EUS in our institution. Of these, 30 (0.77%) had repeat EUS-FNA for the evaluation of possible pancreatic cancer. Two patients were excluded since tissue diagnosis of malignancy was established with the first EUS-FNA, but a repeat study was requested to obtain additional tissue for special staining. The remaining 28 patients (12 male, 16 female), mean age 62 years (range 34–82), were included in the analysis, and the results are reported.

The most common presenting symptoms were abdominal pain (78.57%), weight loss (64.29%), and nausea/vomiting (39.29%) (Table 1). The indication for the initial EUS was abnormal CT or MRI findings in 27 (96.43%) patients and endoscopic retrograde cholangiopancreatography (ERCP) abnormalities in 1 (3.57%). Preprocedure CT or MRI showed definitive or possible pancreatic mass in 26 patients (88.89% and 7.41%, resp.) and no desecrate mass in 1 (3.70%), whose indication for the EUS was common bile duct stricture on ERCP.

Initial EUS evaluation showed pancreatic mass in 24 (85.71%), no mass in 3 (10.71%) patients, and possible mass in 1 (3.58%). FNA was done in all 28 (100%) patients. Initial exam was limited, and less than four FNA passes were made in 5 patients (17.86%) due to difficulty in positioning the echoendoscope or sedating in 3 patients (10.74%), presence of ascites and vascular collaterals in 1 (3.58%), and food obstructing view in 1 (3.58%). The mean number of FNA passes on initial EUS was 4.82 (1–7, range). Cytologic evaluation of the FNA specimen was “atypical” in 1 (3.58%), “suspicious” in 4 (14.28%), and “benign/reactive” in 23 (82.14%). No complications were related to the first EUS.

Repeat EUS was performed in all patients after mean of 33 days (range 2–140 days) from the initial EUS exam. At the second EUS-FNA, a pancreatic mass was seen in 27 patients (96.42%) and no mass in 1 (3.58%). The patient in whom no mass was seen had a distal bile duct stricture and biliary stent. FNA was obtained from the area immediately surrounding the stent. Cytology was positive for malignancy on repeat EUS-FNA in 6 (21.43%) patients. The second EUS established the tissue diagnosis of malignancy in 1 (3.58%) patient with prior atypical/suspicious findings at the first EUS and in 5 (17.85%) of the patients with benign/reactive initial cytology. Based on the location of the lesion, malignancy was diagnosed at the second EUS-FNA in 3 (50%) of the lesions in the head/uncinate process and in 3 (50%) of the lesions in the neck, body, and tail. The mean number of FNA passes on the repeat EUS examination was 4.82 (range 2–8). Overall repeat EUS-FNA established definitive tissue diagnosis of malignancy in 6 out of 28 patients (21.43%). There were not complications related to the second EUS.

Out of the 28 patients in this cohort, 17 (60.71%) were eventually confirmed to have cancer. Tissue sampling confirmed malignancy in 11/17 (64.71%), and clinical criterion (progressing metastatic disease/local invasion on imaging, or death within 1 year upon presentation) established malignancy in 6/17 (35.29%). Of the 11 patients in whom the diagnosis of malignancy was established by tissue pathology, adenocarcinoma was present in 8/11(72.73%), and there was one case of neuroendocrine tumor (9.09%) lymphoma (9.09%) and spindle cell neoplasm (9.09%). Repeat EUS-FNA was able to provide tissue confirmation in 6 (35.29%) of the patients eventually found to have pancreatic malignancy (*n* = 17).

According to our predetermined criteria, 11/28 (39.29%) of the patients were considered to have no malignancy. One (9.09%) was diagnosed with autoimmune pancreatitis, 4 (36.36%) had chronic pancreatitis-associated inflammatory head masses which eventually resolved on followup imaging, 2 (18.18%) had chronic calcific pancreatitis, and in 4 (36.36%) patients no definitive diagnosis was established. Of these 4 patients without established definitive diagnosis but classified as having benign disease, two had a pancreatic mass by both CT and EUS; however, both died from unrelated causes, respectively, two and a half and three years after presentation. The third patient had a suspected pancreatic mass on CT scan, but definitive mass was not seen on EUS. EUS-FNA was repeated total of three times, and all cytology specimens were benign. The patient is still alive and stable over 2.5 years. Lastly, the 4th patient was lost to followup and was considered as having benign disease in this intention to treat analysis. 

Repeat EUS-FNA correctly determined the true final status in 17 out of 28 patients providing a sensitivity for the diagnosis of cancer of 35% (95% CI 14%–62%), specificity of 100% (95% CI 72%–100%), and overall accuracy for determination of the true final status of the patients of 61% (95% CI 28%–72%).

## 4. Discussion

The main finding of our study is that, in patients with suspected pancreatic cancer and negative EUS-FNA, repeat EUS-FNA had a moderate clinical yield. Repeat EUS-FNA was accurate in predicting the final malignant/benign status in 61% of the patients and provided positive tissue diagnosis in 35% of the patients eventually found to have pancreatic malignancy. 

At present, there is no universally accepted strategy on how to manage patients presenting with clinical history and imaging findings suggestive of pancreatic malignancy but who have negative cytology on EUS-FNA. The currently available options consist of clinical observation with serial imaging, CT-guided biopsy, surgical exploration with “blind” pancreatic resection, chemoradiation therapy without definitive tissue diagnosis, and repeat EUS-FNA. Clinical observation is rarely a feasible option due to the high anxiety associated with the potential diagnosis of pancreatic cancer, and CT-guided biopsy has fallen out of favor mostly due to the increased risk of intraperitoneal spread of the cancer via the needle track and worse outcome even in patients that are not surgical candidates [8]. Surgical exploration done purely for tissue diagnosis is rarely carried out in patients who are not candidates for pancreatic resection due to the high degree of invasiveness but can be considered in patients who appear resectable based on imaging and are otherwise fit for surgery. The appeal of “blind” pancreatic resection and chemoradiation therapy without definitive tissue diagnosis has significantly diminished considering the medicolegal climate in the US. Finally, in patients with clinical suspicion for pancreatic malignancy and negative EUS-FNA, one can consider repeating the EUS exam and reattempting FNA. This repeat EUS-FNA strategy is appealing due to the minimally invasive nature of EUS and its excellent safety record, but the data in support of such approach are limited. In patients evaluated with EUS for various indications, DeWitt et al. reported that repeat examination had a clinical impact in 63% of patients [9]. To date only study has specifically evaluated the value of repeat EUS-FNA in patients presenting with suspicion for pancreatic cancer [10]. In these series of 24 patients, the reported accuracy of the repeat EUS-FNA was found to be 84% [10]. The current study expands on these previous findings. Despite our somewhat lower accuracy of 61%, our findings reaffirm that repeat EUS-FNA is a reasonable management strategy. 

Our study is not without limitations. All cases were done in a tertiary referral center by highly experienced endosonographers raising concerns about the applicability of our findings in various practice settings. Furthermore, our patient population is relatively small, and therefore the study did not have enough power to evaluate for any predictors for success/failure for the repeat EUS-FNA. In addition, in our study, we aimed to obtain minimum of 4 FNA passes per patient. Unfortunately, there is no uniform consensus on the optimal number of needle passes needed to obtain correct diagnosis. Earlier findings demonstrated that an average of 3.44 passes provided adequate cytological sampling, but more recent higher quality prospective study revealed that 7 passes are needed to achieve FNA sensitivity of 83% [11, 12]. Finally, we did not have data on the serum levels of immunoglobulin G subclass 4 (IgG4) in most patients since the importance of autoimmune pancreatitis as a cause of pancreatic mass lesions has been just recently fully appreciated [13].

In conclusion, in patients with suspicion for pancreatic cancer and negative EUS-FNA, a repeat EUS-FNA can provide tissue diagnosis in approximately one-third of patients eventually found to have malignancy. Although repeat EUS-FNA provides a relatively low sensitivity and accuracy, it still appears an attractive option given its minimally invasive nature, excellent safety record, suboptimal alternatives for tissue sampling, and the ramifications of a lack of definitive tissue diagnosis.

## Figures and Tables

**Table 1:** Patient presenting symptoms.

Initial presentation of patient	
Asymptomatic	39.29% (*n* = 11)
Jaundice	35.71% (*n* = 10)
Pruritis	3.58% (*n* = 1)
Weight loss	64.29% (*n* = 18)
Abdominal pain	78.57% (*n* = 22)
Nausea/vomiting	39.29% (*n* = 11)
Early satiety	25.00% (*n* = 7)
Lethargy	17.86% (*n* = 5)
Unknown	10.71% (*n* = 3)

## Abbreviations

EUS: Endoscopic ultrasound
FNA: Fine needle aspiration
ERCP: Endoscopic retrograde cholangiopancreatography
CT: Computed tomography
MRI: Magnetic resonance imaging.
